# Factors associated with common mental disorder symptoms and lived experiences among adults with hypertension in Nepal: a mixed-methods study

**DOI:** 10.3389/fepid.2026.1769884

**Published:** 2026-09-14

**Authors:** Lisasha Poudel, Binita Acharya, Bipsana Shrestha, Shreya Shrestha, Archana Shrestha, Buna Bhandari, Brandon A. Kohrt, Dristy Gurung, Sauharda Rai

**Affiliations:** 1 Central Department of Public Health, Institute of Medicine, Tribhuvan University, Kathmandu, Nepal; 2 Transcultural Psychosocial Organization (TPO) Nepal, Kathmandu, Nepal; 3 Institute for Implementation Science and Health, Kathmandu, Nepal; 4 Department of Public Health, Kathmandu University School of Medical Sciences, Dhulikhel, Nepal; 5 Department of Global Health and Population, Harvard T.H. Chan School of Public Health, Boston, MA, United States; 6 Dwyer School of Health Sciences, Indiana University South Bend, South Bend, IN, United States; 7 Center for Global Mental Health Equity, Department of Psychiatry and Behavioral Health, George Washington University, Washington, DC, United States

**Keywords:** anxiety, comorbidity, depression, hypertension, mental health

## Abstract

**Background:**

In hypertension care, common mental disorder (CMD) symptoms such as depression and anxiety shape adherence, self-management, and outcomes. Evidence from low- and middle-income settings on factors associated with CMD symptom severity and patients’ lived experiences remains limited. This study aimed to examine factors associated with depressive and anxiety symptom severity among adults with hypertension in Nepal and to explore how patients’ lived experiences contextualize the quantitative findings.

**Methods:**

We conducted a community-based, explanatory sequential mixed-methods study in Budhanilkantha Municipality (Kathmandu, Nepal). Quantitatively, 327 adults with hypertension were selected via systematic random sampling. Depressive and anxiety symptom severity were measured with Patient Health Questionnaire-9 (PHQ-9) and Generalized Anxiety Disorder-7 (GAD-7) and modeled as **c**ontinuous outcomes using linear regression in prespecified blocks (socio-demographic; health/medication; lifestyle) and a fully adjusted model. Qualitatively, we conducted in-depth interviews with 16 purposively selected participants (with and without depressive/anxiety symptoms). Transcripts were thematically analyzed and interpreted using a Biopsychosocial framework. We integrated quantitative and qualitative findings across conceptually related domains to identify areas of convergence, complementarity, and contextual elaboration.

**Results:**

In fully adjusted models, sleeping ≥6 h per day was associated with lower depressive and anxiety symptom scores. Non-high-caste group was associated with higher depressive symptom scores, whereas older age was associated with lower anxiety symptom scores. Consumption of >2 servings of fruits and vegetables per day was associated with lower depressive symptom scores. Higher household income and controlled blood pressure showed borderline inverse associations with anxiety symptom scores. Qualitatively, hypertensive patients with CMD symptoms reported persistent insomnia, bothersome medication side-effects, activity restriction, loneliness/stigma, anger/fear, low confidence, and financial strain, whereas those without symptoms described adaptation to medicines, stronger family support, and greater self-efficacy. Mixed-methods integration showed strong convergence for sleep, while qualitative findings complemented quantitative patterns related to financial burden and medication experiences and provided contextual elaboration of perceived hypertension symptoms.

**Discussion:**

Sleep duration, social position, age, diet, and socioeconomic circumstances were associated with CMD symptom severity among adults with hypertension in Nepal. The integrated findings highlight the importance of considering sleep, treatment experiences, financial circumstances, and social support within hypertension care.

## 1 Introduction

The global shift in the burden of disease from communicable to non-communicable diseases (NCDs) presents significant public health challenges. Among NCDs, hypertension is a significant contributor to cardiovascular diseases and a leading cause of mortality worldwide (1). It accounts for approximately 7.6 million premature deaths globally, representing about 13.5% of the total mortality (2, 3). Often referred to as the “silent killer,” its asymptomatic nature complicates early detection, making regular monitoring essential for effective management (4). Notably, around 80% of patients with hypertension reside in low- and middle-income countries (LMICs) including our study site Nepal (5).

Psychological distress including mental health conditions is a recognized risk factor for developing hypertension (6). The interplay between hypertension and common mental disorders (CMD) such as anxiety and depression, is increasingly recognized as a critical aspect of hypertension management (7, 8). Studies indicate that patients with hypertension experience elevated levels of CMD compared to the general population, which may hinder their ability to adhere to treatment regimens and manage their condition effectively (9–11). Consequently, patients suffering from both mental health issues and hypertension are more likely to experience uncontrolled Blood Pressure (BP) and increased mortality rates from cardiovascular diseases (12–14). This relationship underscores the importance of addressing psychological factors in the clinical management of hypertension. While psychological interventions have shown promise in reducing blood pressure and improving quality of life (15), there remains a critical gap in understanding the prevalence of anxiety and depressive symptoms among individuals with hypertension, particularly in LMIC (16), where healthcare resources are often limited.

Nepal faces a significant burden of hypertension, affecting one in four adults (17), alongside heterogeneous social conditions and uneven access to care. Yet few studies in Nepal examine factors associated with CMD symptoms (18–20) or integrate patients’ lived experiences to illuminate mechanisms linking social position, daily routines, and clinical care to mental health in hypertension. This study aimed to move beyond prevalence estimates to examine which factors are associated with the severity of depressive (Patient Health Questionnaire- PHQ-9) and anxiety (Generalized Anxiety Disorder-7—GAD-7) symptoms among adults with hypertension in Nepal and how these symptoms are experienced in daily life. Using an explanatory sequential mixed-methods design, we (i) quantified associations between depressive and anxiety symptom severity and prespecified socio-demographic, health/medication, and lifestyle factors; (ii) conducted in-depth interviews with participants with and without elevated CMD symptoms to explore their lived experiences of hypertension and psychological distress, including medication side-effects, sleep, functional limitations, emotions, social support, stigma, and financial strain; and (iii) integrated the quantitative and qualitative findings across conceptually related domains to identify areas of convergence and complementarity, while using the qualitative findings to contextualize rather than causally explain the observed quantitative associations.

## 2 Methods

### 2.1 Study design

We conducted a cross-sectional study using an explanatory sequential mixed methods design (21) that systematically integrated qualitative and quantitative findings. The quantitative phase was conducted first, followed by qualitative interviews with a purposively selected subsample of participants with and without elevated CMD symptoms based on their PHQ-9 and GAD-7 results. Thus, the two phases were connected primarily through participant selection. The qualitative phase explored participants’ lived experiences of hypertension and psychological distress and was used to contextualize quantitative findings where conceptually related information was available. Data was collected from January 2024 to April 2024.

### 2.2 Study site

This study was conducted in Budhanilkantha (Figure 1), an urban municipality in the Kathmandu district. The municipality has a population of 146,828 and is divided into 13 wards, with 11 public health facilities. The prevalence of hypertension in Bagmati province, to which the municipality belongs, is higher than the national average (25.2% vs. 24.5%) (22).

**Figure 1:**
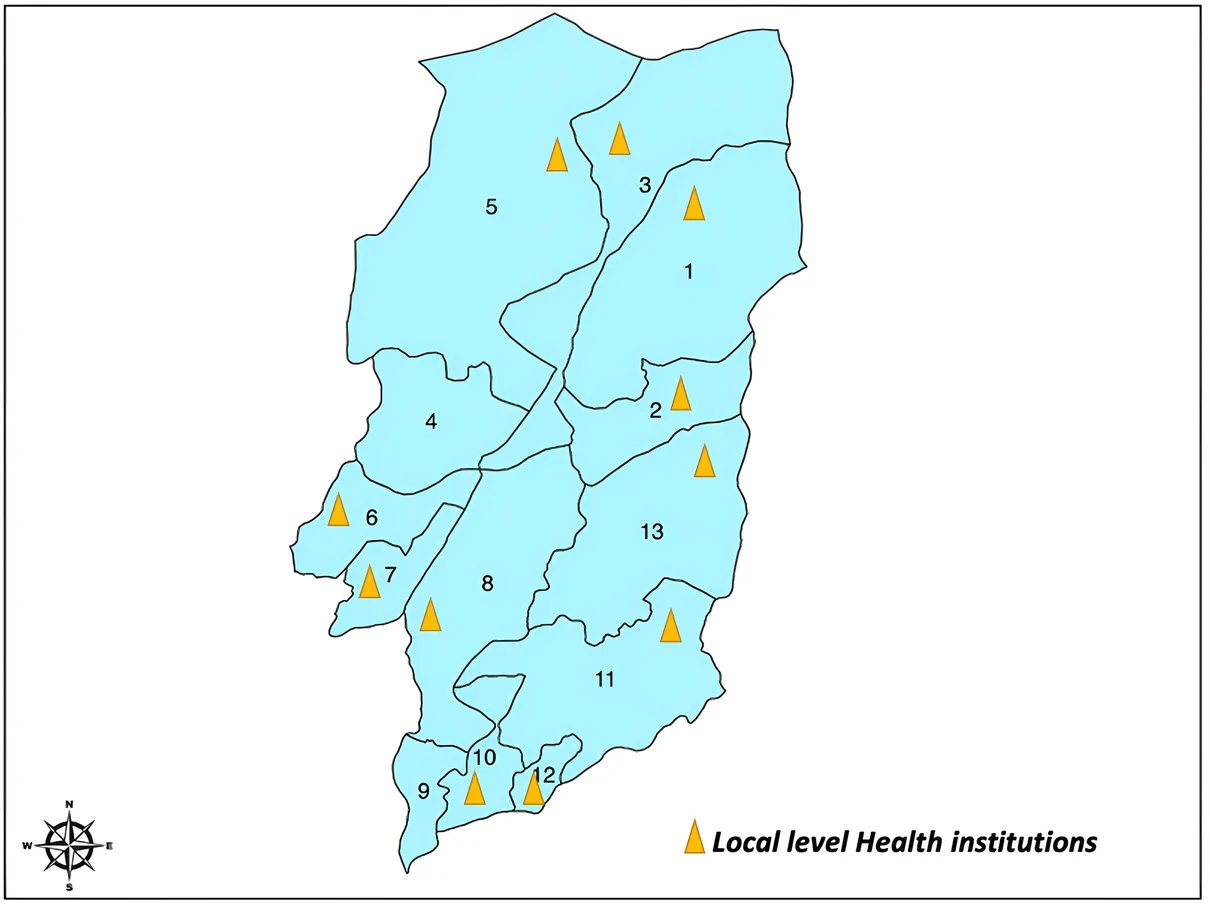
Map of budhanilkantha municipality.

### 2.3 Study population

We selected individuals with hypertension residing in the study site to participate in the study. Participants had to be aged 18 years or older and diagnosed as hypertensive (defined as having a systolic blood pressure of 140 mmHg or more and/or a diastolic blood pressure of 90 mmHg or higher or who were using antihypertensive medication). Participants were required to provide written consent for the study. The study excluded pregnant women and critically ill individuals who were hospitalized or bedridden. The qualitative study included a sub-sample of this population selected on the basis of having or not having CMD symptoms.

### 2.4 Sample size

#### 2.4.1 Quantitative

The sample size was calculated using Cochran's formula (23), with a prevalence of depressive symptoms at 26.8% (24), 5% absolute error, 5% level of significance, and a 10% non-response rate, with a total sample size of 331. A list of 1,253 individuals with hypertension from Budhanilkantha Municipality was used for sampling. A systematic random sampling method was applied, with a calculated interval of every 4th individual (k = 1253/331 ≈ 4) selected for the final sample of 331 participants. STATA version 17 was used for sampling.

#### 2.4.2 Qualitative

We included all patients who experienced CMD symptoms, totaling 8 cases (2 cases experiencing both elevated depressive and anxiety symptoms). For comparison, we also included 8 patients with hypertension who did not experience CMD symptoms. In total, we included 16 patients with hypertension in the qualitative study.

### 2.5 Study variables

#### 2.5.1 Quantitative variables

##### 2.5.1.1 Outcome variables (common mental disorders)

###### 2.5.1.1.1 Depressive symptoms

Depressive symptoms was measured using the PHQ-9, a self-administered tool with nine items to assess symptom severity. Each item is scored from 0 (“not at all”) to 3 (“nearly every day”), yielding a total score between 0 and 27. This study utilized a validated Nepali version of the PHQ-9, with scores of 10 or higher indicating potential major depressive disorder (25).

###### 2.5.1.1.2 Anxiety symptoms

Anxiety was measured using the GAD-7 (26) questionnaire, a self-report tool with seven items that assess anxiety severity. Each item score ranges from 0 (“not at all”) to 3 (“nearly every day”), for a total possible score of 0–21. A Nepali version of the GAD-7 was used in this study, with scores of 9 or higher suggesting the presence of significant anxiety symptoms.

The PHQ-9 and GAD-7 were used to assess depressive and anxiety symptom severity, respectively. The specified cut-off scores were used to identify participants screening positive for elevated symptoms; these screening results were not considered clinical psychiatric diagnoses.

##### 2.5.1.2 Exposure variables

###### 2.5.1.2.1 Socio-demographic characteristics

This included Age(in years), Sex(Female/Male), Ethnicity(high caste/non high caste), Education in years, Marital Status (Married/Single/Widow/Divorced), Occupation(Employed/Unemployed), Monthly income [Nepali Rupees (Nrs) < 50,000, Nrs ≥50,000], Family Support in hypertension management (Yes/No).

###### 2.5.1.2.2 Clinical History

Blood pressure (Controlled/Uncontrolled), Duration since hypertension diagnosis (in years), Currently taking medicine(Yes/No), Duration since medication used (in years), Types of Medicine (27) (Angiotensin receptor blockers/ Calcium channel blockers/Others), Comorbidity (Yes/No), Types of comorbidity (open-ended).

###### 2.5.1.2.3 Behavioural Characteristics

Medication adherence assessed by Morisky Medication Adherence Scale (MMAS-4) (28) (Poor/Good), Smoking (never smoker/former smoker/someday smoker/everyday smoker), Physical activity using International Physical Activity Questionnaire (IPAQ) (29) (Vigorous/Moderate/Low), Diet (>2 servings/<2 servings of fruits and vegetables per day), and Sleep duration (average hours per day).

#### 2.5.2 Qualitative variables

The interview guideline was developed based on the literature (30, 31). The study contained an interview guide [Supplementary file 1: Interview guide] that explored patients’ experiences, including their physical, social, emotional, and financial impact.

### 2.6 Ethical considerations

The Nepal Health Research Council granted ethical approval for the study (Ref no. 746). The research team provided all participants with a detailed explanation of the study's goals, objectives, and risk and benefits of participation. Literate participants provided written informed consent after receiving detailed study information. For participants who were not literate, a witness nominated by the participant provided written consent on their behalf. Study approval was also provided by the local municipality. To protect participants’ privacy, all personal identifiers were removed during data analysis. A unique participant ID code was assigned in place of identifiable information. Only the Principal Investigator had access to the key linking codes and identifiable information, which were stored securely in a password-protected database. All other research staff accessed only de-identified datasets. Throughout the study, strict protocols were followed to maintain the confidentiality and privacy of all participants.

As at least one healthcare worker in each local institution had received training in psychological counseling, we planned to refer participants screening positive for elevated depressive or anxiety symptoms to these facilities for further support.

### 2.7 Data collection

Data was collected in coordination with the local municipality, 11 local health institutions (government), Female Community Health Volunteers (FCHVs), and ward representatives. Three research assistants, each with prior experience in mental health research, were recruited for data collection. They underwent a comprehensive two-day training session focused on the study protocol, ethical considerations, obtaining informed consent, administering the PHQ-9 and GAD-7 questionnaires, and conducting qualitative interviews. The training also included practical exercises, role-plays, and mock interviews to ensure consistency and accuracy in data collection across all study sites.

The list of patients with hypertension was obtained from the municipality after securing formal approval from the health section of the municipality. These records included patients with hypertension who had visited local health facilities such as Health Posts, Primary Health Care Centers, Basic Health Service Centers, and Urban Health Centers. The list of patients with hypertension was generated using systematic random sampling and included contact information (names, addresses, and phone numbers). Selected participants were initially contacted by phone to arrange in-person interviews conducted at their homes. Initially, 331 individuals were selected. Of these, 62 could not be reached by telephone and 9 declined participation. To achieve the required sample size, replacement participants were selected by continuing through the original sampling frame using the same systematic sampling interval until the target number of participants was reached.

Data was collected using the KOBOToolbox. Blood pressure measurements were taken using an Omron digital device, with three readings taken at five-minute intervals. The average of the last two readings was calculated to classify blood pressure as controlled or uncontrolled. Participants exhibiting depressive or anxiety symptoms were referred to local health facilities for follow-up care. Emergency contact information (such as a suicide helpline and police and fire department numbers) was included in the informational flyer provided to all participants during the consent process.

Participants who completed the quantitative survey including the PHQ-9 and GAD-7—were considered for qualitative interviews based on their symptom scores. Scoring of the PHQ-9 and GAD-7 was conducted only after the full completion of quantitative data collection. Following this, participants identified with and without major CMD symptoms were re-contacted to schedule a qualitative interview at a time convenient for them. During these follow-up calls, participants were informed that they had been selected for an additional in-depth interview to explore their experiences related to mental health and hypertension. All selected participants provided informed consent for the qualitative interviews, resulting in a 100% consent rate. Each participant was contacted individually to arrange a suitable time for the in-depth interview (IDI). Interviews lasted between 30 and 35 min, were audio-recorded with prior consent, and subsequently transcribed and translated for analysis. No compensation was provided to participants for their involvement in the study.

### 2.8 Data analysis

We used STATA version 17 for data analysis. After excluding missing data (1.2%), a total of 327 observations were included in the analysis. Descriptive statistics were used for participant characteristics. Categorical variables were presented numerically as percentages and frequency. The mean and standard deviation (SD) were reported for continuous data that follow a normal distribution. For continuous data that did not follow a normal distribution, the median and interquartile range (IQR) were reported. The confidence interval around the proportion was determined using the Clopper-Pearson method.

To assess associations with common mental disorder (CMD) symptom severity, we fit ordinary least squares linear regressions with PHQ-9 (depression) and GAD-7 (anxiety) scores as continuous outcomes, reporting unstandardized *β* coefficients with 95% confidence intervals (CIs). Robust standard errors were used in all regression models to account for potential heteroskedasticity. Linearity and influential observations were assessed using residual diagnostics, and multicollinearity was evaluated using variance inflation factors (VIFs). We first estimated domain-specific models for (a) socio-demographic, (b) health/medication, and (c) lifestyle variables, then fit a fully adjusted model including all predictors. Although validated PHQ-9 and GAD-7 cut-off scores were used to describe the proportion of participants with clinically relevant symptoms and to inform purposive selection for the qualitative phase, the total PHQ-9 and GAD-7 scores were treated as continuous symptom-severity outcomes in the regression analyses. Retaining the full scores preserved variability in symptom severity and avoided the loss of information that would result from dichotomizing the outcomes. Categorical explanatory variables were entered into the regression models using indicator (dummy) variables, with one category designated as the reference group; they were therefore not treated as continuous variables. Continuous explanatory variables were retained in their continuous form unless otherwise specified.

For qualitative analysis, all interviews were transcribed, and the interviewer and a trained research assistant reviewed the transcripts for accuracy against the audio recordings. Thematic analysis (32) was utilized for data analysis. This process involved becoming familiar with the data, generating initial codes, identifying themes, reviewing themes, defining and naming themes, and producing the report. The research team developed a hierarchical codebook by synthesizing codes derived directly from the data (inductive) and from the interview guides (deductive). After coding the initial transcripts, the two researchers met to compare the labels they had applied and agreed upon the codes to be applied to all subsequent transcripts with 80.4% inter-coder agreement. The codes were then categorized broadly and grouped under common themes, which were reviewed and defined. The themes, categories, codes, and relevant quotes were translated into English. The Biopsychosocial Model (33), which integrates biological, psychological, and social factors in understanding health and illness, guided the analysis and conceptualization of the data to understand the patients with hypertension experience with CMD symptoms. After organizing the data into matrices and synthesizing them into broader themes, the patient experiences were integrated into a Biopsychosocial Model to better illustrate the findings (Figure 2). Following separate quantitative and qualitative analyses, the two strands were integrated by comparing findings across conceptually related domains. Integration focused on identifying convergence, complementarity, and contextual elaboration. Qualitative findings were used to contextualize quantitative patterns.

**Figure 2:**
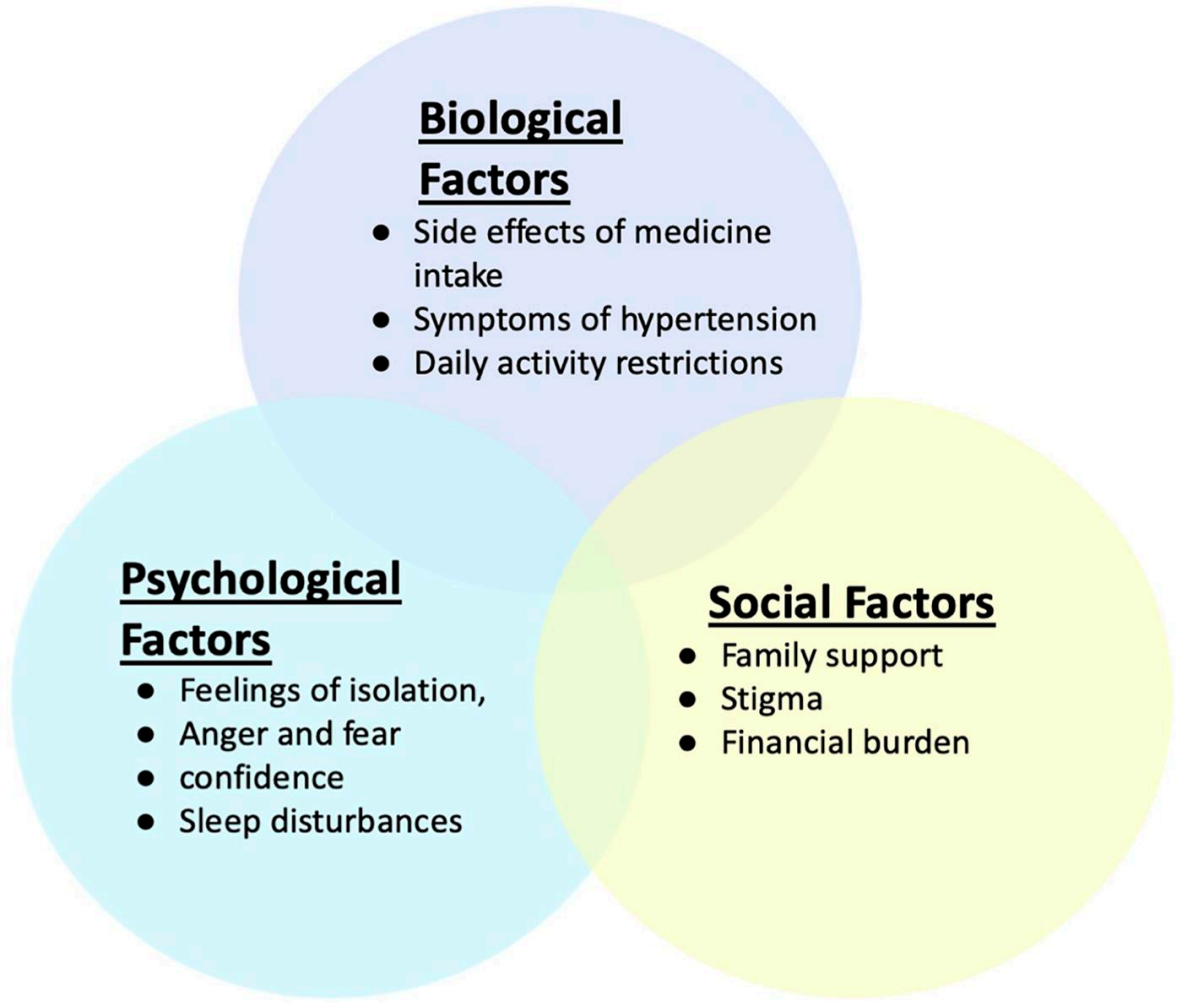
Patient experiences with CMD symptoms using biopsychosocial model.

## 3 Results

### 3.1 Quantitative findings (*n* = 327)

#### 3.1.1 Participant characteristics

##### 3.1.1.1 Socio-demographic characteristics

The study included 327 patients with hypertension, predominantly female (56.57%) and slightly older, with more than half of the participants over 55 years old (55.35%) and a mean age of 58.08 ± 12.29 years. The majority of the participants were from non-high caste groups (54.43%), had at least some education (61.77%) with a mean of 5.55 ± 2.25 years of schooling, and were married (88.69%). A significant portion of the study population was unemployed (57.19%), and nearly half (45.57%) reported a family income of less than 50,000 NRs per month and the majority (97.86%) of participants reported positive family support (Table 1).

**Table 1:** Characteristics of participants (*n* = 327).

Variables	n (%)
Socio-demographic Characteristics	
Gender	
Male	142 (43.43)
Female	185 (56.57)
Age in years (Mean ± SD)	58.08 ± 12.29
30–55 years	146 (44.65)
>55 years	181 (55.35)
Ethnicity	
High caste	149 (45.57)
Non-high caste	178 (54.43)
Education in years (Mean ± SD)	5.55 ± 2.25
No education	125 (38.23)
Any education	202 (61.77)
Marital Status	
Single	5 (1.53)
Married	290 (88.69)
Divorced	1 (0.31)
Widow	31 (9.48)
Occupation	
Employed	140 (42.81)
Unemployed	187 (57.19)
Family Income (Monthly)	
<50,000 NRs	149 (45.57)
≥50,000 NRs	178 (54.43)
Family Income in NRs (Median, IQR)	55,000 (40,000)
Family Support	
Yes	320 (97.86)
No	7 (2.14)
Clinical history of participants	
Blood Pressure	
Controlled	151 (46.17)
Uncontrolled	176 (53.83)
Duration since hypertension diagnosis (Mean ± SD)	7.79 ± 6.79
<5 years	165 (50.46)
6–10 years	81 (24.77)
More than 10 years	81 (24.77)
Currently Taking Medicine	
Yes	319 (97.55)
No	8 (2.45)
Duration since Medication Used (Mean ± SD) (*n* = 319)	7.63 ± 6.78
1–10 years	243 (76.18)
More than 10 years	76 (23.82)
Types of Medicine (*n* = 319)*	
ARBs (Angiotensin Receptor Blockers)	130 (40.75)
Calcium Channel Blockers	264 (82.87)
Others	28 (8.77)
Comorbidity	
Yes	131 (40.06)
No	196 (59.94)
Major Diseases (*n* = 131)*	
Cancer	2 (1.52)
Diabetes	68 (51.90)
Chronic obstructive pulmonary disease (COPD)	10 (7.63)
Heart Disease	3 (2.29)
Thyroid	18 (13.74)
Others	22 (16.79)
Behavioral characteristics of study participants	
Medication adherence (*n* = 319)	
Good	249 (78.06)
Poor	70 (21.94)
Currently smoking	
No	281 (85.93)
Yes	46 (14.07)
Cigarettes per day (Mean, SD)	7.8 ± 6.71
Diet (Fruits and vegetables)	
>2 servings	79 (24.16)
<2 servings	248 (75.84)
Sleep (average hour per day) (Mean, SD)	7.07 ± 1.18
< 6 h	25 (7.65)
6 h and more	302 (92.35)
Physical Activity	
Low physical activity	86 (26.30)
Moderate physical activity	217 (66.36)
Vigorous physical activity	24 (7.34)

##### 3.1.1.2 Clinical characteristics

Among the 327 participants, 151 (46.17%) had controlled blood pressure, and the mean duration since hypertension diagnosis was 7.79 years. A total of 319/327 participants (97.55%) were taking antihypertensive medication, with a mean duration of medication use of 7.63 years. Among those taking medication (*n* = 319), 264 (82.87%) used calcium channel blockers. Comorbidity was present in 131/327 participants (40.06%). Among participants with comorbidity (*n* = 131), diabetes was the most common condition, reported by 68 (51.90%).

##### 3.1.1.3 Behavioral characteristics

A majority (78.06%) showed good adherence, 14.07% were current smokers (averaging 7.8 cigarettes/day), 75.84% ate less than two servings of fruits/vegetables daily, with an average sleep of 7.07 h per day, and 26.30% reported low physical activity.

#### 3.1.2 Prevalence of screening-positive depressive and anxiety symptoms

Among the 327 adults with hypertension, 5 (1.53%; 95% CI: 0.50%–3.53%) screened positive for depressive symptoms based on the PHQ-9 cut-off, and 5 (1.53%; 95% CI: 0.50%–3.53%) screened positive for anxiety symptoms based on the GAD-7 cut-off. Two participants (0.61%; 95% CI: 0.07%–2.19%) screened positive for both depressive and anxiety symptoms.

#### 3.1.3 Factors associated with depressive and anxiety symptoms

##### 3.1.3.1 Factors associated with depressive symptoms

Table 2 presents domain-specific and fully adjusted linear models of depressive symptom severity (PHQ-9). In socio-demographic models (Model 1), non-high caste participants had higher PHQ-9 scores by 1.8 points compared with high caste (*β*=1.80, 95% CI: 0.45–3.10), while older age was associated with slightly lower scores (*β*=−0.03, 95% CI: −0.06–0.001). Income ≥50 K showed a borderline inverse association (*β*=−0.64, 95% CI: −1.30–0.05). In health/medication models (Model 2), comorbidity was associated with higher PHQ-9 (*β*=0.69, 95% CI: 0.21–1.20), whereas blood-pressure control, medication adherence, and duration measures were not associated. In lifestyle models (Model 3), sleep ≥6 h was strongly associated with lower PHQ-9 (*β*=−2.80, 95% CI: −3.70 to −2.00); diet >2 servings/day and higher physical activity trended toward lower scores but were not statistically significant.

**Table 2:** Factors associated with depressive symptoms.

Characteristic	Model 1			Model 2			Model 3			Model 4		
	Beta	95% CI^a^	*p*-value	Beta	95% CI^1^	*p*-value	Beta	95% CI^a^	*p*-value	Beta	95% CI^a^	*p*-value
Gender												
Female	–	—										
Male	0.39	−0.37, 1.1	0.3							−0.25	−1.0, 0.51	0.5
Age												
<55 years	-											
≥55 years	−0.03	−0.06, −0.001	0.033							−0.03	−0.06, 0.001	0.069
Ethnicity												
High caste	—	—								—	—	
Non-high caste	1.8	0.45, 3.1	0.009							1.8	0.59, 3.1	0.004
Income												
Less than 50K	—	—								—	—	
50 K and above	−0.64	−1.3, 0.05	0.071							−0.75	−1.6, 0.06	0.068
Education												
No education	—	—								—	—	
Any education	−0.35	−1.2, 0.50	0.4							−0.46	−1.3, 0.36	0.3
Blood Pressure												
Uncontrolled				—	—					—	—	
Controlled				−0.12	−0.61, 0.36	0.6				−0.20	−0.91, 0.50	0.6
Comorbidity												
No				—	—					—	—	
Yes				0.69	0.21, 1.2	0.005				0.38	−0.28, 1.0	0.3
Medication adherence												
Low				—	—					—	—	
High				−0.55	−1.1, 0.04	0.068				−0.40	−1.3, 0.48	0.4
Duration of taking meds												
1–10 years				—	—					—	—	
More than 10 years				−0.55	−3.6, 2.5	0.7				−0.56	−4.6, 3.5	0.8
Duration of Hypertension												
< 5 years				—	—					—	—	
6–10 years				0.66	−2.3, 3.7	0.7				0.88	−3.1, 4.9	0.7
More than 10 years				0.58	−2.5, 3.7	0.7				0.43	−3.7, 4.5	0.8
Diet												
< 2 serving							—	—		—	—	
> 2 serving							−0.41	−1.0, 0.17	0.2	−1.0	−2.0, −0.05	0.040
Smoking												
No							—	—		—	—	
Yes							0.24	−0.40, 0.88	0.5	−0.68	−1.6, 0.27	0.2
Physical Activity												
Low							—	—		—	—	
Moderate							−0.40	−0.98, 0.17	0.2	−0.35	−1.3, 0.58	0.5
High							−0.84	−1.8, 0.13	0.091	−0.76	−2.4, 0.87	0.4
Hours of sleep												
< 6 h							—	—		—	—	
6 h and more							−2.8	−3.7, −2.0	<0.001	−3.2	−4.4, −2.1	<0.001

a CI, confidence interval.

The fully adjusted model (Model 4), which included all socio-demographic, health/medication, and lifestyle variables, showed three independent associations. First, non-high-caste remained associated with higher PHQ-9 (*β*=1.80, 95% CI: 0.59–3.10). Second, sleeping ≥6 h was associated with substantially lower PHQ-9 (*β*=−3.20, 95% CI: −4.40 to −2.10). Third, diet >2 servings/day was associated with lower PHQ-9 scores(*β*=−1.00, 95% CI: −2.00 to −0.05). Age (*β*=−0.03, 95% CI: −0.06–0.001) and income ≥50 K (*β*=−0.75, 95% CI: −1.60–0.06) showed borderline inverse associations. The associations for gender, education, blood-pressure control, medication adherence, comorbidity, and duration of hypertension/medication were not statistically significant after full adjustment. The association between comorbidity and PHQ-9 observed in Model 2 attenuated after adjustment and was no longer statistically significant in Model 4.

##### 3.1.3.2 Factors associated with anxiety symptoms

Table 3 presents domain-specific and fully adjusted linear models of anxiety symptom severity (GAD-7). In socio-demographic models (Model 5), older age was associated with lower GAD-7 (*β*=−0.05; 95% CI −0.07 to −0.02; *p* = 0.001), and income ≥50 K was linked to lower scores (*β*=−0.96; 95% CI −1.60 to −0.34; *p* = 0.002). Gender, education, and ethnicity were not associated. In health/medication models (Model 6), comorbidity related to higher GAD-7(*β*=0.47; 95% CI 0.05–0.90; *p* = 0.028) and high medication adherence to lower GAD-7 (*β*=−1.10; 95% CI −1.60 to −0.62; *p* < 0.001), while blood-pressure control and duration variables showed no associations. In lifestyle models (Model 7), sleep ≥6 h was strongly associated with lower GAD-7 (*β*=−2.60; 95% CI −3.30 to −1.80; *p* < 0.001); smoking, physical activity, and diet were not significant, although diet >2 servings/day trended toward higher scores (*β*=0.50; 95% CI −0.04–1.00; *p* = 0.067). The fully adjusted model (Model 8), which included all socio-demographic, health/medication, and lifestyle variables, retained two independent associations. Sleep ≥6 h remained associated with lower GAD-7 scores (*β*=−2.60; 95% CI −3.70 to −1.60; *p* < 0.001), and older age continued to relate to lower GAD-7 (*β*=−0.05; 95% CI −0.07 to −0.02; *p* < 0.001). Income ≥50 K (*β*=−0.69; 95% CI −1.40–0.04; *p* = 0.065) and controlled blood pressure (*β*=−0.60; 95% CI −1.20–0.05; *p* = 0.070) showed borderline inverse associations. There were no independent associations for gender, education, ethnicity, comorbidity, medication adherence, smoking, physical activity, or the duration variables (all *p* ≥ 0.2). The associations for comorbidity and medication adherence observed in Model 6 attenuated after adjustment and were no longer statistically significant in Model 8.

**Table 3:** Factors associated with anxiety symptoms.

Characteristic	Model 5			Model 6			Model 7			Model 8		
	Beta	95% CI^a^	*p*-value	Beta	95% CI^a^	*p*-value	Beta	95% CI^a^	*p*-value	Beta	95% CI^a^	*p*-value
Gender												
Female	—	—								—-	—	
Male	0.19	−0.48, 0.87	0.6							−0.35	−1.1, 0.34	0.3
Age												
<55 years	—-	—-								—-	—-	
≥55 years	−0.05	−0.07, −0.02	0.001							−0.05	−0.07, −0.02	<0.001
Ethnicity												
High caste	—	—								—	—	
Non-high-caste	0.75	−0.42, 1.9	0.2							0.82	−0.32, 2.0	0.2
Income												
Less than 50K	—	—								—	—	
50 K and above	−0.96	−1.6, −0.34	0.002							−0.69	−1.4, 0.04	0.065
Education												
No education	—	—								—	—	
Any education	0.13	−0.62, 0.89	0.7							−0.01	−0.76, 0.74	>0.9
Blood Pressure												
Uncontrolled				—	—					—	—	
Controlled				−0.27	−0.70, 0.15	0.2				−0.60	−1.2, 0.05	0.070
Comorbidity												
No				—	—					—	—	
Yes				0.47	0.05, 0.90	0.028				0.38	−0.21, 0.98	0.2
Medication adherence												
Low				—	—					—	—	
High				−1.1	−1.6, −0.62	<0.001				−0.17	−0.97, 0.64	0.7
Duration of taking meds												
1–10 years				—	—					—	—	
More than 10 years				−0.30	−2.9, 2.4	0.8				0.21	−3.5, 3.9	>0.9
Duration of Hypertension												
< 5 years				—	—					—	—	
6–10 years				0.88	−1.8, 3.5	0.5				0.51	−3.2, 4.2	0.8
More than 10 years				0.20	−2.5, 2.9	0.9				0.03	−3.7, 3.8	>0.9
Diet												
< 2 serving							—	—		—	—	
> 2 serving							0.50	−0.04, 1.0	0.067	0.05	−0.85, 0.94	>0.9
Smoking												
No							—	—		—	—	
Yes							0.22	−0.36, 0.80	0.5	−0.48	−1.4, 0.39	0.3
Physical Activity												
Low							—	—		—	—	
Moderate							−0.37	−0.89, 0.15	0.2	−0.35	−1.2, 0.51	0.4
High							−0.42	−1.3, 0.46	0.3	−0.45	−1.9, 1.0	0.5
Hours of sleep												
< 6 h							—	—		—	—	
6 h and more							−2.6	−3.3, −1.8	<0.001	−2.6	−3.7, −1.6	<0.001

a CI, confidence interval.

### 3.2 Qualitative findings (*n* = 16)

The qualitative component included a total of 16 participants, comprising two groups: hypertensive patients with common mental disorder (CMD) symptoms (H-CMD, *n* = 8) and hypertensive patients without CMD symptoms (H-noCMD, *n* = 8). Detailed participant characteristics are presented in the Supplementary file-2.

#### 3.2.1 Biological factors

##### 3.2.1.1 Side effects of medicine intake

Participants in both groups reported adverse effects after initiating antihypertensive medication, but their experiences differed in persistence and perceived impact. H-CMD participants more often described ongoing dizziness, headache, weakness, and sleep disturbance, sometimes accompanied by reluctance to take medication regularly. In contrast, H-noCMD participants generally described side effects as temporary or manageable, with symptoms improving over time or after medication or dose adjustments.

“When I first started taking the medicine, it was really hard because I had a lot of side effects like stomach pain, vomiting, and diarrhea. It made me feel very uncomfortable. But now, those problems have gone away, and I’m feeling much better.” —**H-noCMD_5, Male, 55 years.**

“After being diagnosed with hypertension, my doctor initially prescribed a high-dose medication. I experienced severe headaches and body pain, so I consulted my doctor again. He then switched me to a different medication with a lower dose, and since then, I haven't experienced any side effects.” —**H-noCMD_3, Male, 37 years**

These reflects that treatment experiences were perceived as more disruptive among participants with elevated CMD symptoms, whereas participants without CMD symptoms more commonly described adaptation to treatment.

##### 3.2.1.2 Physical health issues of hypertension

Participants in both groups described physical symptoms that they attributed to hypertension, although the nature and interpretation of these symptoms varied. Headache was common across both groups. H-CMD participants more frequently described symptoms such as dizziness, blurred vision, fatigue, sweating, and lethargy alongside emotional distress. H-noCMD participants tended to emphasize physical complaints such as chest discomfort, muscle pain, neck discomfort, and difficulty walking.

“It feels like something is pressing heavily on my eyes as well. It’s like I’m feeling dizzy too. Even when I tilt my head to the side, I feel as if something is really pressing down on my eyes, making it hard to see.” **—H-CMD_4, Female, 45 years**

“Sometimes, when my blood pressure goes up, I feel a lot of pain in my legs and muscles…. It gets so bad that I can’t walk easily. I also notice that my whole-body hurts, making it really uncomfortable to do anything.” **—H-noCMD_8, Female, 53 years**

This indicate that perceived physical illness burden was present in both groups, but among H-CMD participants physical symptoms were more often described together with psychological distress.

##### 3.2.1.3 Daily activity restriction

Functional limitations were reported by both groups. Participants described difficulty completing household tasks, working, walking, or maintaining previous levels of physical activity. H-CMD participants more frequently linked these restrictions with fatigue, poor concentration, breathlessness, and reduced motivation, while H-noCMD participants more often attributed limitations to aging, body weight, or physical health.

“I've been feeling short of breath lately when I try to do a bit more work. I can’t manage to do a lot of tasks, whether it’s because I’m overweight or just feeling exhausted.”**—H-CMD_6, Female, 36 years**

“It might be because of my age; I can't do much anymore. Even going to buy things for the house has become difficult for me. Daily tasks feel really hard due to my age.” **—H-noCMD_7, Female, 75 years**

Thus, activity restriction was not unique to participants with CMD symptoms, but its emotional and functional consequences appeared more pronounced among H-CMD participants.

#### 3.2.2 Psychological factor

##### 3.2.2.1 Feeling of isolation and loneliness

Social isolation emerged as a prominent experience among H-CMD participants. They frequently described feeling lonely or misunderstood even when family members or friends were physically present. Some reported withdrawing from social interactions because others did not recognize or understand their distress.

“I often feel very lonely…This feeling of isolation is heavy on me and makes it difficult to do everyday tasks or enjoy being around my family. Because of this, I struggle to find the energy or desire to do anything, whether it’s helping out around the house or just spending time with my loved ones.” **—H-CMD_2, Female, 55 years**

In contrast, H-noCMD participants generally described regular interaction with family and friends and did not report persistent loneliness.

“I have a family who supports me at home and friends outside whom I hang out with, so I don't feel lonely.”**—H-noCMD_3, Male, 37 years**

##### 3.2.2.2 Anger and fear

H-CMD participants commonly reported irritability, sudden anger, fear, and emotional distress. Anger was sometimes directed toward family members and followed by guilt or regret. Some participants also described feeling frightened or startled without an identifiable reason. Fear was not exclusive to the H-CMD group. Some H-noCMD participants also expressed fear, particularly regarding possible complications or death from hypertension. However, among H-CMD participants, fear was more often accompanied by broader emotional distress and difficulties regulating mood.

“When things go wrong at home, I feel anger toward whoever caused the problem. It really bothers me, and I often end up yelling at my son and granddaughter. Even though I care about them, my frustration sometimes takes over, and I forget to be patient. After I cool down, I realize that I shouldn’t have reacted that way, and it makes me feel guilty for losing my temper. My son d often tells me not to let little things upset me so much. He thinks my anger adds to my stress, and he’s right.”**—H-CMD_5, Male, 52 years**

“I often have severe headaches. I've heard people say that those with hypertension have fainted and even died while going to the toilet. I get scared about what might happen to me, especially after experiencing many headaches.”**—H-noCMD_8, Female, 53 years**

##### 3.2.2.3 Confidence

A marked contrast was observed in participants’ perceived confidence in managing daily life and their health. Several H-CMD participants described low self-confidence, reduced motivation, and feelings of inadequacy. One participant stated:

“My confidence is extremely low, and I feel like I can’t do anything in life. After being affected by this disease, I don’t even have the motivation to accomplish anything.”**—H-CMD_4, Female, 45 years**

In contrast, H-noCMD participants more often expressed confidence in their ability to manage their condition and continue with daily activities.

“My confidence is actually very high; everything is fine. I’m from the mountains. I bought land in Kathmandu, and built a house this is because i was confident from the very first.” **—H-noCMD_5, Male, 55 years**

This difference shows that reduced self-efficacy was an important feature of the lived experience among participants with elevated CMD symptoms.

##### 3.2.2.4 Sleep disturbance

Sleep disturbance was one of the clearest differences between the two groups. H-CMD participants frequently described persistent insomnia, daytime sleepiness, and long-term use of sleeping medication. Some reported continued difficulty sleeping despite taking medication.

“I can't sleep at night if I don't take my medication. I have to take sleeping pills to be able to sleep, and it’s been like this for 6–7 years.” **—H-CMD_2, Female, 55 years**

H-noCMD participants generally described satisfactory sleep, although occasional sleep difficulties were reported.

“Sometimes, I don't get enough sleep, but most of the time, my sleep is good.” **-H-noCMD_5, Male, 55 years**

#### 3.2.3 Social factor

##### 3.2.3.1 Family support

Some H-CMD expressed a lack of family support, noting that their children live abroad, leaving them alone without any assistance. Additionally, a few reported a lack of support from their husbands, which arose from ongoing conflicts in their relationships. This situation contributed to heightened anxiety and distress for these individuals.

“I don't have any family support; my sons are abroad, and my daughters are already married. I live with my husband, but we fight most of the time. I feel like a burden to him, especially due to my illness, which irritates him sometimes and i fear to lose him.”**—H-CMD_8, Female, 54 years**

H-noCMD reported having strong family support in terms of disease management, financial assistance, and emotional encouragement.

“I have good family support….my son and daughter-in-law take good care of me. They take me to the hospital when I am sick or need follow-up, and they also take me out sometimes when I feel low.” -**H-noCMD_7, Male, 75 years**

##### 3.2.3.2 Stigma

Experiences of stigma and exclusion were primarily described by H-CMD participants. Some reported being treated differently because of hypertension, being viewed as physically incapable, or feeling excluded from social activities. One participant also described concealing medication use in social settings because of concerns about how others perceived his illness.

“At work, sometimes they go out drinking after work, and I feel left out because I can't drink much….people look at me differently, saying that my blood pressure and sugar levels are the reason for my condition. This makes me feel bad. In front of them, I avoid taking my medication and have said that my hypertension is getting better, even though it actually hasn’t..and” **—H-CMD_5, Male, 52 years**

“At my home, when the family members come, they say, ‘You have high blood pressure; you can't do anything.”**—H-CMD_2, Female, 55 years**

These accounts indicate that perceived stigma extended beyond the physical effects of hypertension and influenced participants’ social interactions and willingness to disclose their condition.

##### 3.2.3.3 Financial burden

All H-CMD and some H-noCMD reported experiencing a financial burden related to managing their hypertension. Many expressed difficulties affording the necessary medications, and even though they receive an elderly allowance, it often falls short of covering their healthcare needs.

“I dont have money to treat my disease… I need to take medicine daily, and with the current high prices, I can't afford to buy it. My son isn't earning anything either. It’s really difficult to manage financially.” **—H-CMD_2, Female, 55 years**

Some H-noCMD shared that they receive financial support from family members, which helps alleviate some of the financial pressure associated with managing their condition.

“I've always had enough because of God’s grace…. I have enough money… My daughter and sons provide me with the money for my disease management…”**—H-noCMD_5, Male, 55 years**

### 3.3 Mixed method integration

Mixed-methods integration focused on domains for which the quantitative and qualitative strands provided conceptually related information. Qualitative findings were used to contextualize and elaborate quantitative patterns rather than to infer causal mechanisms. Table 4 summarizes the principal areas of convergence, complementarity, and contextual elaboration.

**Table 4:** Integrating qualitative and quantitative data .

Domain	Quantitative finding	Qualitative elaboration	Integrated interpretation
Sleep	Sleeping ≥6 h/day was associated with lower PHQ-9 (*β*=−3.20, *p* < 0.001) and GAD-7 scores (β=−2.60, *p* < 0.001) in fully adjusted models.	Participants with CMD symptoms frequently described persistent insomnia, daytime sleepiness, and reliance on sleeping medication, whereas those without CMD symptoms generally reported satisfactory sleep.	Strong convergence. Both strands consistently identified sleep disturbance as closely related to greater CMD symptom burden; however, directionality cannot be established.
Income/financial burden	Higher income was associated with lower anxiety scores in the socio-demographic model and remained borderline after full adjustment (β=−0.69, *p* = 0.065).	Participants with CMD symptoms commonly described difficulty affording medicines and treatment, while some without CMD symptoms reported financial support from family.	Complementarity. Qualitative accounts contextualized the socioeconomic pattern observed quantitatively, without establishing financial strain as a causal mechanism.
Medication adherence/treatment experience	Higher adherence was associated with lower GAD-7 scores in the health/medication model (β=−1.10, *p* < 0.001), but the association was not retained after full adjustment.	Participants with CMD symptoms described dizziness, headaches, and other medication-related discomfort and sometimes reluctance to take medication; participants without CMD symptoms more often described adaptation or improvement after medication changes.	Complementarity. Interviews provide context about treatment experiences that may accompany adherence difficulties but do not explain the quantitative association causally.
BP control/perceived symptoms	BP control was not independently associated with depressive symptoms and showed only a borderline inverse association with anxiety in the fully adjusted model (β=−0.60, *p* = 0.070).	Participants described headaches, dizziness, fatigue, pain, and fear that they attributed to hypertension regardless of measured BP control.	Contextual elaboration. Participants’ perceived illness burden remained important even when measured BP control was not clearly associated with CMD symptom severity.

CMD, common mental disorder; PHQ-9, patient health questionnaire-9; GAD-7, generalized anxiety disorder-7; BP, blood pressure; β, unstandardized regression coefficient. Quantitative estimates are from the respective regression models. Qualitative findings contextualize or elaborate quantitative patterns and should not be interpreted as evidence of causal mechanisms.

## 4 Discussion

This mixed-methods study examined factors associated with CMD symptom severity and lived experiences of common mental disorder (CMD) symptoms among adults with hypertension in an urban Nepalese municipality. Sleep duration showed the strongest association with both depressive and anxiety symptom scores. This is consistent with previous research linking mental health issues to both short and long sleep durations (34–37). Interviews provided granular detail: participants with elevated symptoms described persistent insomnia, reliance on sleep medicines for years, and ongoing non-restorative sleep despite pharmacotherapy. Participants without CMD symptoms more often reported satisfactory sleep. Adequate sleep is crucial for regulating metabolism and maintaining quality of life (38). Reduced sleep duration and quality have been associated with alterations in endocrine and immune functioning and with poorer mental and physical health outcomes (38, 39). However, given the cross-sectional design, the direction of this association remains uncertain, and residual confounding from unmeasured factors cannot be excluded; therefore, further longitudinal studies are needed to clarify the relationship between sleep and CMD symptoms.

The study found a significant association between ethnicity and depressive symptoms in individuals with hypertension, with non-high caste participants such as Dalits, a socially marginalized group, showing higher depressive symptoms scores than those from higher social strata. This aligns with previous research highlighting that ethnicity influences depression and hypertension in developing Asian countries (40–42). A community-based study conducted in Nepal demonstrated a higher prevalence of depression among low-caste groups (43). This association was linked to various factors, including exposure to stressful life events, household income, and livestock ownership (43). Likewise, studies on African Americans have shown that racial discrimination and social inequities contribute to increased depressive symptoms, earlier onset of chronic diseases, higher cardiovascular risk, and poorer self-rated health (44–47). Qualitative accounts provided additional context regarding participants’ social experiences, including loneliness, social exclusion, stigma, and financial strain. However, these experiences were not specifically examined as mechanisms underlying the association between caste/ethnicity and depressive symptoms. Taken together, the findings highlight the potential importance of social and economic disadvantage in the broader experience of psychological distress among adults with hypertension. These findings support consideration of equity-sensitive approaches to hypertension care, including respectful communication, attention to financial barriers, and strengthening family and social support where appropriate.

Concurrently, the inverse association between higher income and anxiety symptom scores corroborates the well-established socioeconomic gradient in mental health, consistent with prior studies (20, 48). The qualitative findings complement this quantitative pattern. Participants with CMD symptoms frequently described economic hardship, particularly difficulties affording medicines and treatment, whereas some participants without CMD symptoms described financial support from family members. These accounts provide contextual information about the socioeconomic circumstances experienced by participants but do not establish financial strain as a causal mechanism underlying anxiety symptoms. Together, the findings indicate that economic circumstances are relevant to the broader context in which psychological distress is experienced.

Diet >2 daily servings of fruits/vegetables were associated with lower depressive symptoms scores but not for anxiety. While our diet measure was coarse, it is directionally consistent with literature linking diet quality to depressive symptoms via inflammation, microbiome, and energy regulation pathways (49, 50). Blood pressure control was not independently associated with CMD severity, yet in qualitative interview, participants frequently described bodily symptoms and fears that they attributed to hypertension. These qualitative findings provide context regarding participants’ perceived illness burden but do not directly explain the quantitative findings related to measured BP control.

Our qualitative findings reveal that patients with hypertension with depressive and anxiety symptoms experience significant psychological stress. Many participants expressed feelings of anger, fear, and lack of confidence, which exacerbated their mental health challenges. These emotions often arise from the stress of managing a chronic condition like hypertension. Even patients without depressive and anxiety symptoms voiced fears of death. A qualitative study on hypertension patients found that fear and concern were common sentiments, particularly among those who had directly or indirectly faced complications from high blood pressure (51, 52). Other studies (52, 53) also indicated that patients experienced fear and concern regarding hypertension complications, aligning with our findings.

While this study is among the first to examine CMD symptoms among individuals with hypertension in Nepal using both quantitative and qualitative methods, it has several limitations. The cross-sectional nature of the quantitative component precludes causal interpretation of the observed associations and does not allow the temporal direction of relationships between participant characteristics and depressive or anxiety symptom severity to be established. Reverse causality and residual confounding from unmeasured factors also cannot be excluded. Additionally, participants were selected through systematic random sampling from a municipality-maintained list of individuals with hypertension. Therefore, individuals who had not sought healthcare, had received care exclusively from private facilities, or were not recorded in the municipal database were not represented. Replacement of non-responders may also have introduced selection bias, as individuals who could not be contacted or declined participation may have differed from those who participated in healthcare engagement, socioeconomic characteristics, or mental-health symptoms. The use of self-reported measures for CMD symptoms and medication adherence may have introduced recall and social desirability bias. Finally, the qualitative interviews were designed primarily to explore participants’ lived experiences rather than to directly investigate mechanisms underlying specific quantitative associations. Accordingly, the integrated findings should be interpreted as providing convergence, complementarity, or contextual elaboration where conceptually related information was available, rather than as evidence of causal pathways.

## 5 Conclusions

In adults with hypertension in Nepal, sleep, social position, age, diet, and socioeconomic context were associated with CMD symptom severity. Mixed-methods integration showed particularly strong convergence regarding sleep, while qualitative findings provided additional context concerning financial burden, medication experiences, perceived symptoms, and social experiences. These findings support consideration of sleep assessment, attention to medication-related concerns, social support, and financial barriers within hypertension care. Longitudinal and intervention studies are needed to determine directionality and evaluate whether addressing these factors improves mental health outcomes.

## Data Availability

The raw data supporting the conclusions of this article will be made available by the authors, without undue reservation.
