# Supplementary material for: Factors associated with common mental disorder symptoms and lived experiences among adults with hypertension in Nepal: a mixed-methods study
Source: Front Epidemiol. 2026 Sep 14;6:1769884. doi: 10.3389/fepid.2026.1769884 (PMC13617016; doi:10.3389/fepid.2026.1769884)
Supplement: Supplementary file 1 [file Table1.pdf]

In-depth Interview Guide

Ensure recorder is on
Informed consent is received

Introduction

Thank you very much for your participation in the interview that took place a few days ago. The insights shared were truly valuable. Today, we want to hear from you about your experience with hypertension, and its physical, social, emotional and financial impact.

**Information about the interview**	
Date of interview:	
Participant ID:	Interviewer ID:
Start time:	End time:

**Questions**	**Probe**
Hypertension-related characteristics	
Can you tell me in detail of how you came to know that you have high blood pressure and what you have been doing about it since then?	
How do you perceive the reasons behind your high blood pressure?	
How serious do you consider your hypertension?	- Why do you think that?
What changes have occurred in your life or health habits following the diagnosis of hypertension?	
Physical Impact	
Could you please share your experience regarding any symptoms you may have noticed related to hypertension?	- symptoms that are you currently having,<br>- any symptoms when you first took the medication
How was your personal experience with BP-lowering medication(s)?	● any side effects<br>● measures taken to reduce the side effect(s) by you and/or doctor(s)<br>● the positive aspects of taking BP-lowering medication(s)
Can you share how hypertension has influenced your daily activities and routines?	Activity restriction:<br>- Sleep disturbance<br>- Daily work hampered
How do you manage the physical impacts of high BP?	- Eg: doctor consultation, exercise<br>- Did it work ?

Social Impact	
Could you elaborate on any social changes you've experienced as a result of hypertension	Such as any changes in:<br>- your work, job performance, or relationships with your colleague<br>- your life plan, and personal interests<br>- experience discrimination<br>- family/friends relationships (Networking<br>- sexual activity
How do you cope with these social challenges?	- Eg: communication<br><br>What specific strategies have you found helpful?
Emotional Impact	
How has hypertension affected you emotionally? Can you share any specific emotional challenges or changes you've encountered?	- Eg: Self confidence, Fears, Loneliness, loss of control
How do you deal with and manage these problems?	Did these coping strategies work for you?
Financial Impact	
How has hypertension impacted your financial situation?	- For Eg: Medication purchase, doctor consultation fees
Can you discuss how you manage financial challenges related to your hypertension?	
We are now at the end of the conversation. Do you have anything else you would like to share on the topics we discussed today?	

Thank you for time !
