# Supplementary material for: Factors associated with common mental disorder symptoms and lived experiences among adults with hypertension in Nepal: a mixed-methods study
Source: Front Epidemiol. 2026 Sep 14;6:1769884. doi: 10.3389/fepid.2026.1769884 (PMC13617016; doi:10.3389/fepid.2026.1769884)
Supplement: Supplementary file 2 [file Table2.pdf]

Table 5. Characteristic of patients with hypertension (n=16, H-CMD= 8, H-noCMD=8)

**Characteristics**	**H-CMD**<br>**n (%)**	**H-noCMD**<br>**n (%)**
**Age, in years (Mean, SD)**	**51.6, 12.01**	**49.12, 13.08**
**Sex**		
Male	1 (12.5)	2 (25.0)
Female	7 (87.5)	6 (75.0)
**Ethnicity**		
Brahmin/Chettri	2 (25.0)	3 (37.5)
Dalit	4 (50.0)	2 (25.0)
Janajati	2 (25.0)	3 (37.5)
**Monthly income (NPR)**<br>**Mean, SD**	32500, 17525.49	54375, 18212.53
**Blood pressure**		
Controlled	4 (50.0)	4 (50.0)
Uncontrolled	4 (50.0)	4 (50.0)
H-CMD: Hypertensive Patients with Common Mental Disorders Symptoms<br>***H-noCMD: Hypertensive Patients without Common Mental Disorders Symptoms***		
